# Banana Plant Disease Classification Using Hybrid Convolutional Neural Network

**DOI:** 10.1155/2022/9153699

**Published:** 2022-02-23

**Authors:** K. Lakshmi Narayanan, R. Santhana Krishnan, Y. Harold Robinson, E. Golden Julie, S. Vimal, V. Saravanan, M. Kaliappan

**Affiliations:** ^1^Department of Electronics and Communication Engineering, Francis Xavier Engineering College, Tirunelveli, India; ^2^Department of Electronics and Communication Engineering, SCAD College of Engineering and Technology, Tirunelveli, India; ^3^School of Information Technology and Engineering, Vellore Institute of Technology, Vellore, India; ^4^Department of Computer Science and Engineering, Anna University Regional Campus, Tirunelveli, India; ^5^Department of Artificial Intelligence and Data Science, Ramco Institute of Technology, Rajapalayam, India; ^6^Department of Computer Science, College of Engineering and Technology, Dambi Dollo University, Dembidolo, Ethiopia

## Abstract

Banana cultivation is one of the main agricultural elements in India, while the common problem of cultivation is that the crop has been influenced by several diseases, while the pest indications have been needed for discovering the infections initially for avoiding the financial loss to the farmers. This problem will affect the entire banana productivity and directly affects the economy of the country. A hybrid convolution neural network (CNN) enabled banana disease detection, and the classification is proposed to overcome these issues guide the farmers through enabling fertilizers that have to be utilized for avoiding the disease in the initial stages, and the proposed technique shows 99% of accuracy that is compared with the related deep learning techniques.

## 1. Introduction

Agriculture is the crucial resource of food for mankind, and it is one of the important factors that decide the economy of a country. Agriculture is considered the main source of income for most developing countries. One of the important parts of the global agro-business is the banana cultivation or banana industry because bananas are rich in minerals such as calcium, manganese, potassium, magnesium, and iron. As this particular crop is having these many vitamins, they are consumed by people all over the world as banana is considered an instant energy booster. As per the statistics from Wikipedia, about 15% of the global banana productions are exported to western countries for consumption. As per the production and export statistics of bananas, about 25.7% of the global banana production is from India, and other major producers of bananas are the Philippines, Ecuador, Indonesia, and Brazil giving a combined contribution of about 20% of the global banana production. The United States is the leading importer of bananas of about 18% of the global imports. The impact of the banana tree getting infected due to disease and due to other climatic changes will cause even 100% loss in the overall countries' banana production and export. Generally, bananas are affected by four major that are black Sigatoka, fusarium wilt colloquially called Panama wilt, Xanthomonas wilt, and bunchy top virus. The details of the various commonly found diseases along with the disease symptoms, appearance, and effects of the disease when it is present as an infection in the banana leaf is described below.

### 1.1. Banana Leaf Disease

The banana crops are all affected by various diseases. The symptoms are visible in leaf, stem, flower, fruit, roots, and suckers. The major diseases that affect the leaf are Xanthomonas wilt, fusarium wilt, black and yellow Sigatoka.

#### 1.1.1. Xanthomonas Wilt

**Figure 1:**
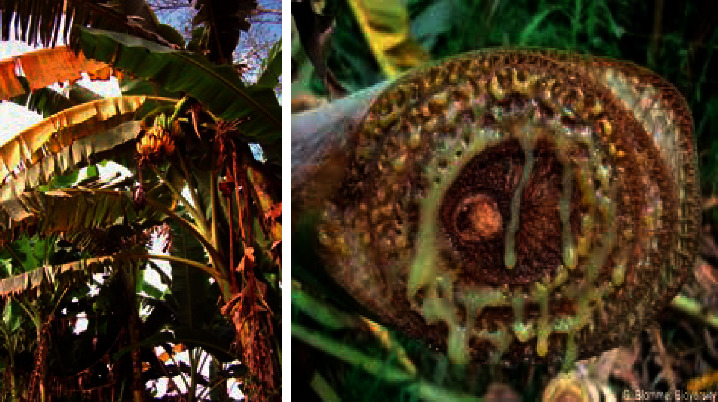
Banana Xanthomonas wilt.

Banana Xanthomonas wilt (BXW) is a bacterial infection caused by *Xanthomonas campestris*. Among the numerous diseases infecting banana, the damage caused by BXW has been huge. The production of bananas is decreased by 30–52% due to BXW. The infected plant appears in pale yellow-orange color and in the later stage it becomes dark brown color. At last, it leads to death, and this may cause 100% yield loss if it is not managed well [1]. The remedy for clearing this disease is by field sanitation and removal of affected plant parts or by spraying streptocycline 200 ppm after the first visual symptom for every 10 days. Further by spraying 0.3% copper oxychloride, it checks the further spread and is demonstrated in Figure 1.

#### 1.1.2. *Fusarium* Wilt

**Figure 2:**
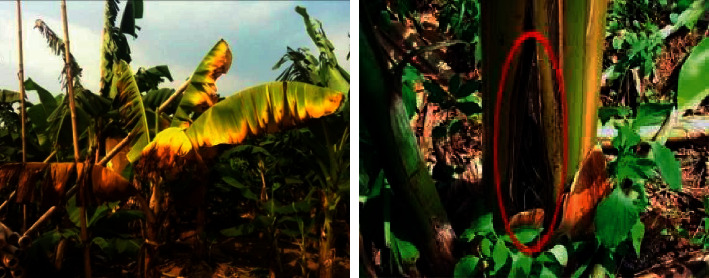
Banana fusarium wilt.

It is usually identified as Panama infection, which is a deadly fungal infection originated from the soil-borne fungus *Fusarium oxysporum*. This disease can cause 100% yield loss if it is not identified and managed in the early stage [2]. The fungus penetrates the plant into the root and settles the xylem vessels, thus jamming the water flow. It appears like a pale yellow color in the early stage and in the later stage, it looks dark in color. It is irregular in shape, pale margin on new leaves, leaf blades are distorted. *Fusarium* wilt is a disease that the fungus assaults the vascular tissue within the root discoloration. The fruit does not show any symptoms. The first sign of the disease is wilting and produces the yellow color of the older leaf at the margin. The tear of the base is the main effect that the infected sucker does not show any symptoms until 4 months. Suggested remedies for this disease infection are applying 2.5 kg/ha Pseudomonas fluorescent in the farmyard along with regular manure and it is illustrated in Figure 2.

#### 1.1.3. Bunchy Top Virus

**Figure 3:**
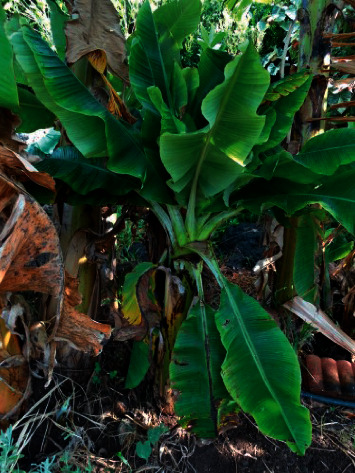
Banana bunchy top virus.

It is a plant pathogenic virus ancestor nanoviridae known for contaminating banana plants. It is a viral infection caused by a single-stranded DNA virus known as the banana bunchy top virus. This disease is affected in a tropical region and transmitted from plant to plant. Symptoms for this infection commonly occur in old plants in which the latest leaves are narrow yellow than normal [3], and the banana bunchy top virus is illustrated in Figure 3. The common remedies for this disease are by injecting 4 ml of fernoxone solution along with 400 ml of water or by injecting 4 ml of monocrotophos on a 1 : 4 ratio at 45 days interval till flowering from the 3rd month. And also by spraying either 1 ml of phosphamidon or 2 ml of methyldemeton or 1 ml of monocrotophos along with water can avoid the disease spread.

#### 1.1.4. Black Sigatoka

**Figure 4:**
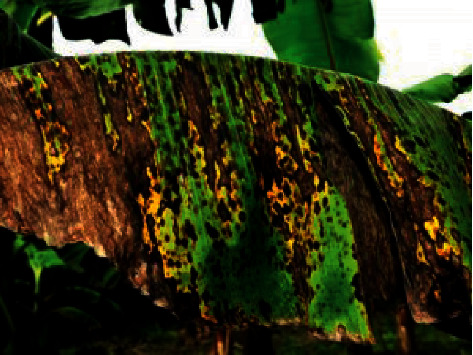
Banana with black Sigatoka.

It is called a black leaf streak caused by mycosphaerella Fijians is that the Sigatoka infection multifaceted is a bunch of intimately correlated fungi. Leaves with huge infectious lesions will begin to collapse, and it interrupts performing photosynthesis, which leads to the death of plants. In the early stage of infection, the lesions have a rusty brown look. After further development, they become darker and turn into depression, which is demonstrated in Figure 4.

This may decrease the yield by 30 to 50% depending upon the severity of the disease infection [4]. The common remedies for this disease are by spraying 3 times with carbendazim or propicanozole or mancozeb and tempol in proper proportion at 10–15 days period, as the disease starts from the original look of leaf specks. Therefore, the detection of the pest and disease at an earlier stage is very important. The traditional methods used for the identification of pests and diseases are limited by the lack of human knowledge based on agriculture [5], so deep learning-based hybrid CNN and FSVM are developed for detecting and classifying the above mentioned common disease found in the banana leaf. This helps in detecting the disease and classifies its type in its early stage [6]. Through this banana, production can be effectively managed.

The rest of the paper is ordered in such a way that related works in the field of plant disease detection that is carried out in Section 2, materials and methods with the proposed method of detecting the banana leaf detection is proposed in Section 3, and Section 4 discusses the results and discussion with a comparison of the proposed system with the existing system, and finally conclusion and future discussion in Section 5.

The main objectives of the proposed model are

1. The main objective of this work is to apply deep learning methodology for the detection of evident banana disease in the banana plant
2. To classify the type of disease with high accuracy
3. By creating a database of insecticides for respective pests and diseases, it will provide a remedy for the disease that is detected
4. As CNN and SVM are the best classifiers available with a hybrid model of combining both the algorithms to produce accelerating performance

## 2. Related Works

The Banana leaf disease detection and classification is an issue for a very long time many kinds of research were done on this field which gave truthful results details of a few such findings were given below.

An artificial intelligence-based banana disease and pest detection was proposed in [7], here the algorithm used is deep convolutional neural network for disease detection, here the author has collected data sets for about 8 different diseases in banana, a sum of 30,000 images were used as a data set, and the proposed system produces a result of 90% accuracy. Machine learning algorithms were developed for detecting and classification of plant diseases [5], reviews various machine learning and deep learning algorithms for detection and classification of plant diseases, and this also identifies some research gaps for detecting the disease in the plants even before the symptoms are visualized.

A deep leaning-based banana leaf disease classification is proposed [8], here they have used LeNet architecture as a convolutional neural network for classifying the data. The results of this research demonstrate its effectiveness in various conditions of the images such as complex background, different size, and orientation. This method gets stabilized in 25 iterations and achieves a good accuracy at the final iteration. A machine learning-based approach for detection of banana disease detection in the early stage using the SVM classifier is proposed [9]. Here the images used are close-range hyperspectral remote sensing images. The results of the classifiers are evaluated by overall accuracy, and average accuracy here is the accuracy using spectral and morphological information, which is about 96% in early detection, 90% in mid detection, and 92% in late detection.

An image processing-based banana leaf disease detection is proposed in [10], here the images are first acquired and the RGB model is converted into an HSI color model and then preprocessed, then the image is segmented using the thresholding method, and the histogram equalization is found for HSI image. Then the classification is compared with three different classifiers such as they are backpropagation neural networks, SVM and principle component analysis (PCA). An artificial neural network-based banana leaf disease detection and classification of the disease is proposed in [11], here the image is acquired and preprocessed initially and then the color and HOT (Histogram of Template) feature are extracted, then the data set is trained using the artificial neural network, and then the grading is done on for the query images based on the total percentage of the affected area. And finally, the image is classified by its disease type.

Not only for banana leaf but also more research is going on for detecting and classifying the disease in most consumable crops like paddy, maize, apple, cheery, and other common plants. Some of those findings are given below. Classification of apple plant and cherry plant diseases using improved convolutional neural networks is proposed in [12]. The improvement in the CNN is based on merging a framework of inception functionality squeeze, excitation functionality, and a global pooling layer. The results of this method show an accuracy of about 91.7% on the test data set. A deep convolutional neural networks and object detection technique based on tomato disease identification have been implemented with two different techniques as Faster R–CNN, which is used for identifying the type of tomato disease and Mask R-CNN, Which is used to find and segment the location and shape of the infected areas, and the results show that the proposed method gives an accuracy of 90% in detecting the disease and 99% in identifying the shape of the infection [13].

Automatic detection and classification of diseases in rice crop are proposed in [14], and here they use an artificial neural network-based technique to identify the disease, they have done research on many diseases that affects the rice crop and measured the accuracy of classifier for each type of disease, and finally they have compared the ANN technique with other leading classifiers for detecting the same diseases and their accuracy were measured and concluded that ANN gives a satisfactory result when compared with the other classification algorithms. A novel rice blast recognition technique related to CNN is proposed in [15]. This method was tested with various combinations such as CNN only, CNN with SVM, LBPH with SVM, and Haar-WT with SVM and their accuracy is compared, and it shows that the CNN with SVM gives an accelerating accuracy of about 96% AUC curve that shows 0.99. The drawbacks of the existing works are

  The main drawback of the existing system is that most of the works follow image processing techniques that involve complex image segmentation steps, which is a very time-consuming process
  Many diseases will not show their symptoms by having a clear edge, and they may merge with the healthy parts of the leaf, which cannot be detected using the existing techniques and requires a powerful classification algorithm
  Certain techniques that are available will be suitable for a particular type of crop or limited to certain crops only
  Methods such as ANN, KNN, PCA, and other image processing techniques are lagging in accuracy and mostly consume more time to classify the disease
  In most cases, the infections in the banana plant will occur in various parts of the plant, but most of the research concentrates only on the leaf

## 3. Materials and Methods

### 3.1. Dataset Collection

The dataset for this proposed research consists of around 3500 images of banana plants, both infected and healthy parts of banana plants were collected from various fields located in south India, especially the southern part of Tamil Nadu from the districts of Madurai, Dindigul, Virudhunagar, Tirunelveli, Tuticorin, Nagercoil, Kanyakumari, and minor parts of Tamil Nadu Kerala border. The data sets were collected in a balanced number of images for 4 basic diseases that can influence the productivity of banana production. The images were collected in various resolutions captured using mobile phones with good resolutions, captured using VGA cameras in mobile, Digital cameras, and DSLR cameras.

### 3.2. Image Preprocessing

**Figure 5:**
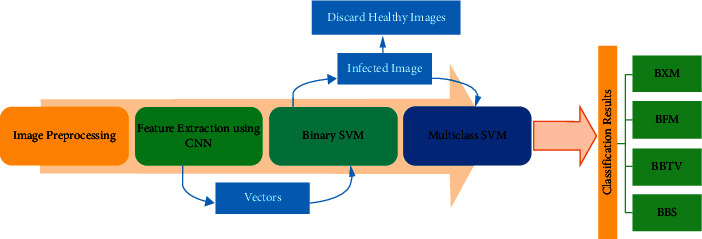
Block diagram of the proposed method.

**Figure 6:**
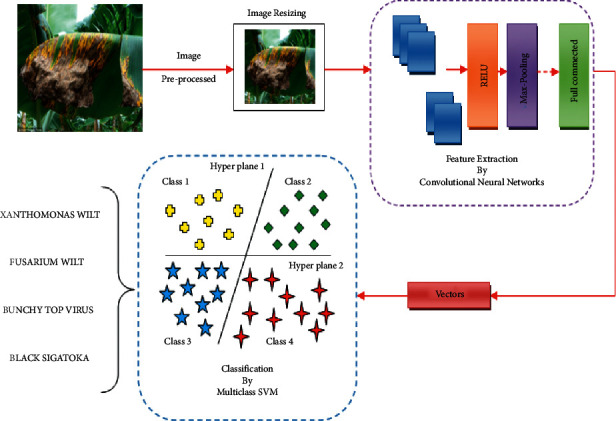
Overview of the proposed method.

Image preprocessing on the collected image is performed to enhance the image quality for performing the further steps. This process does not change the default information in the image; it performs image resizing and performs some useful filtering processes to detect the disease information in the banana leaf. The image captured and stored as a data set is of different resolutions so it must be converted into a standard fixed resolution size using image resizing. Then by using image filtering techniques such as median filter other noises present in the images are removed. Focus issues and other unwanted portions in the image while capturing are restored in this process. Another filtering process such as low pass filtering that helps in reducing the amplitude of high-frequency components in the image and keeps the low-frequency information as it is. The high-pass filter does the vice versa and the proposed architecture is illustrated in Figures 5 and 6.

### 3.3. Feature Extraction Using CNN

**Figure 7:**
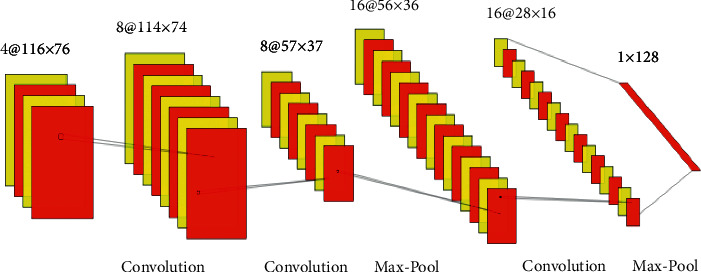
Architecture of the convolutional neural networks for the proposed system.

The main objective of the CNN is to recover the high-level features of the banana leaf infections in the image. To perform this, CNN architecture was built with multiple overlying convolutional layers. Normally, a convolutional layer will contain convolution and activation functions that are non-linear in our case, and the utilization of ReLU and pooling process have been enabled. The input for the CNN will be training and testing images of dimensions of 80 × 120 in height and width, which is demonstrated in Figure 7.

The proposed CNN architecture is motivated by LeNet-5. The convolutional layers are called C1, C2 which contains 4, 8, and 16 filters with dimension 5 × 5, 3 × 3 and 2 × 2, respectively. Let *v*^*l*^ denotes the output of the convolutional layers, and it is expressed in

(1) $$\begin{array}{c} v^{l}=f\left(a^{l}+\overset{X}{\underset{x}{\sum }}\overset{Y}{\underset{y}{\sum }}g_{x,y}^{l}h_{x,y}^{l-1}\right). \end{array}$$

Here,

 
*X*, *Y* represents size of the filters (height and width)
 
*a*^*l*^ represents bias of the convolutional layer
 
*h*^*l*−1^ represents the output of the preceding convolutional layer
 
*g*^*l*^ represents the weight of the convolution layer

The non-linear activation function of ReLU (*f*(*v*)) is computed in

(2) $$\begin{array}{c} f\left(v\right)=ReLU\left(v\right)=f\left(v\right)=\left\{\begin{array}{c} v,\;v>0,\\ 0,\;v\leq 0. \end{array}\right.. \end{array}$$

The parameter *δ* could be computed using the highest estimation with the particular training set, and it is computed in

(3) $$\begin{array}{c} E\left(\delta \right)=\overset{N}{\underset{n=1}{\prod }}f\left(v\right)\delta_{n}, \end{array}$$

where *E*(*δ*) is the highest estimation parameter, and the vector values are computed using

(4) $$\begin{array}{c} \omega^{n}=\overset{n}{\underset{i=1}{\sum }}\omega_{i}^{n}. \end{array}$$

The next operation is pooling that reduces dimensions of the feature maps, and this helps in reducing the computations to be performed in the network. So further operations will be performed on the précised positioned features generated by the convolutional layer. There are multiple types of pooling methods available in this context of the Max pooling method so that the output of the Max-pooling layer will contain the most important features of the earlier feature map. This pooling-based reduction is done without losing the features or patterns.

**Figure 8:**
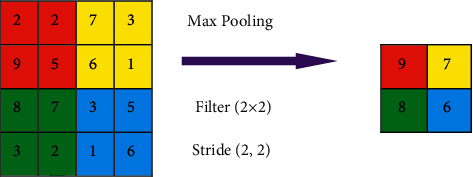
2D Max pooling operations in Keras.

After completing a sequence of convolution layers, ReLU activation, and pooling operations, the next step is to perform the flattening operation that converts the 2D matrix of features into a vector of features, which is then fed into a classifier model. The main objective of the fully connected process is to feed the flattened vectors into the classifier, and the 2D Max pooling operations in Keras are demonstrated in Figure 8.

In this proposed work, train and test the images in various proportions they are 90%, 70%, 50%, 30%, and 20% as training images, and the remaining images are treated as the test images. With these proportions, compare the performance of classification on training accuracy and testing accuracy.

### 3.4. Proposed Fusion SVM Classifier

SVM is a supervised machine learning algorithm and is one of the most popular approaches for image classifications. SVM is a binary classification algorithm that classifies the images into only two distinct classes, infected or not infected. But the real-world problem is to identify the exact infection type, which involves the classifier to classify the image into more than two classes or multiple classes. There are many types of methods that can be followed to use SVM for multiclass classification [16] such as One versus All, One versus One, and All versus All.

**Figure 9:**
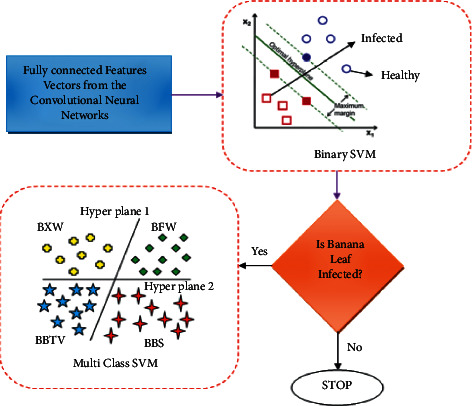
Structure of proposed fusion SVM classifier.

In this proposed work, the SVM is used in two different phases P1 and P2. In the first phase P1, the feature extracted from the CNN model is fed as an input to a binary SVM model. Here the model classifies the leaf image and other parts of the banana plant available in the data set as infected on a healthy leaf. If the test image of the leaf given as an input is a healthy leaf, then the process terminates. In the second phase, P2 construct a multiclass support vector machine to predict the disease type. Given a new image, our proposed model determines whether the banana plant is infected or not. If it is an infected image, then it predicts its class out of 4 available disease classes. The proposed classification method is described in Figure 9.

## 4. Results and Discussions

The testing of the banana tree disease detection and classification was performed using PYTHON programming language in Jupyter notebook environment on an I5 processor with 32 GB of RAM equipped with 6 GB AMD GPUs from NVIDIA. To demonstrate the proposed method of image classification, the experimentation has been conducted with 5 categories of images taken. The proposed system takes the image data and extracts the image features using the proposed CNN whose architecture is inspired by LeNet-5. Then with the extracted features of the fusion-based SVM testing operation, the first-level P1 binary SVM classifies the image as infected leaf or a healthy leaf. If the given test image is a healthy image, the process exits in the phase P1, and the classifier performs the training process when a new image is given as a query image for performing itself. If the test image is infected, then the features are applied to the second phase P2, which is a multiclass classification. Here the infected banana plant is classified as either one of the four classes, namely banana Xanthomonas wilt (BXW), banana *Fusarium* wilt (BFW), banana bunchy top virus (BBTV), and banana black Sigatoka (BBS). Then the performance of the proposed classifier is measured according to the accuracy in percentage and elapsed time (ET) in seconds. Simultaneously, a confusion matrix is constructed to calculate the classification precision, recall, and F1 score.

**Figure 10:**
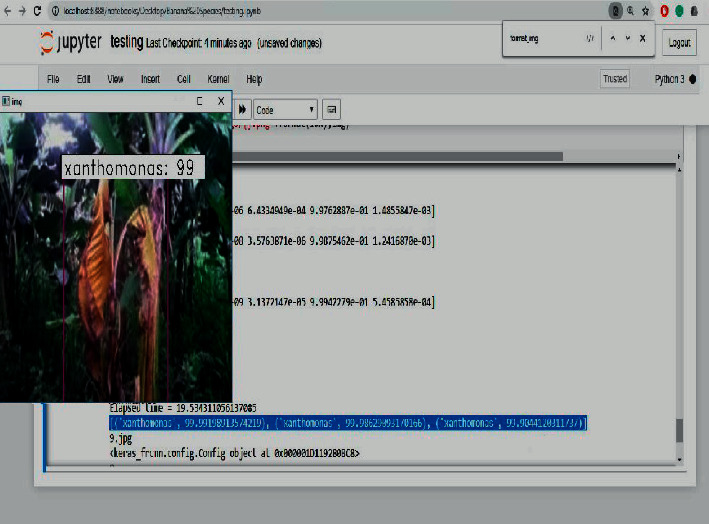
Image classified as BXW showing the accuracy and elapsed time.

**Figure 11:**
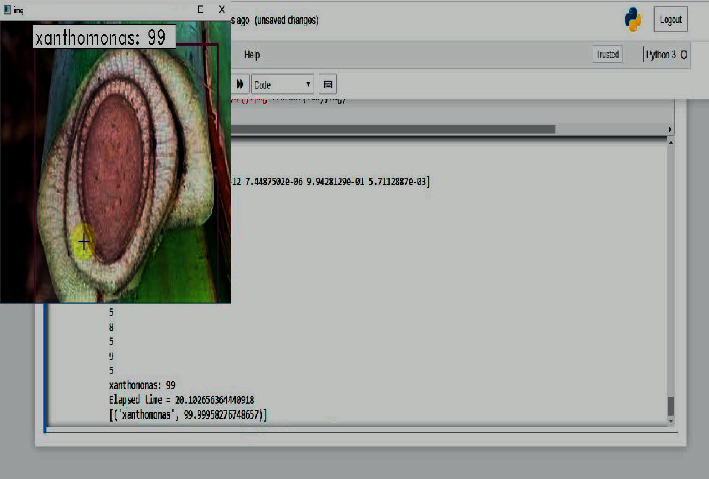
Stem image classified as BXW showing the accuracy and elapsed time.

**Figure 12:**
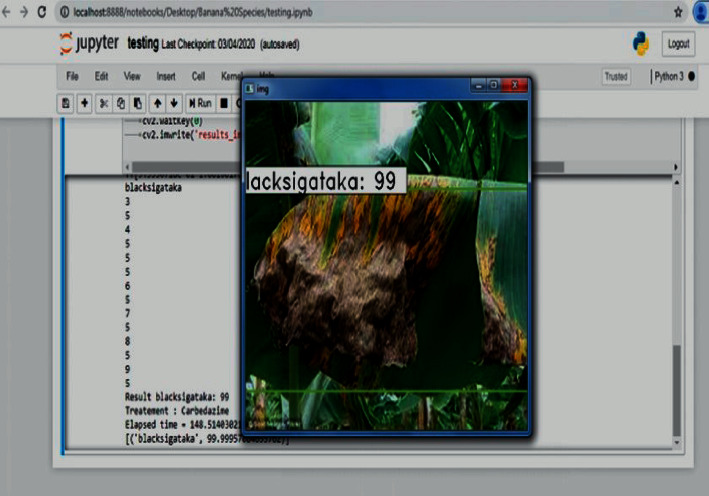
Image classified as BBS showing the accuracy and elapsed time.

Figure 10 shows the output of the proposed system with a classification accuracy of 99% and elapsed time of 19.53 seconds. Furthermore, this output suggests the possible treatments to be given for the detected disease such as if the disease is black Sigatoka, the treatment is Carbedazime; if the predicted disease is Xanthomonas, the treatment is to spray Mancozeb; if the predicted disease is fusarium, the treatment is to spray Propiconazole, and if the predicted disease is bunchy top, Fernoxone is the possible treatment.  Figure 11 shows the query image classified as black Sigatoka with an accuracy of 99% and elapsed time of 148.51 seconds.  Figure 12 illustrates the image classified as BBS showing the accuracy and elapsed time.

**Figure 13:**
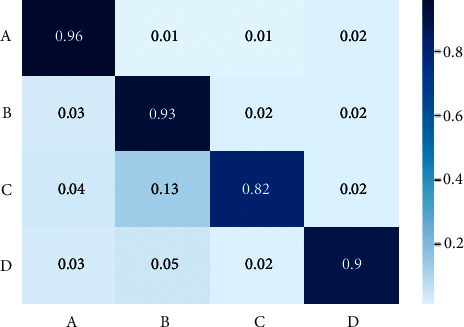
Confusion matrix.

To measure the performance of the model proposed a confusion matrix is constructed. The confusion matrix is a tabular outline representing the efficiency of the proposed model and how it performs.  Figure 13 shows the confusion matrix of the proposed model, there are 4 elements used in the confusion matrix they are.

  True positive (TP): proposed model predicts true, while actually it is true
  True negative (TN): proposed model predicts false, while actually it is false
  False positive (FP): proposed model predicts true, while actually it is false
  False negative (FN): proposed model predicts false, while actually it is true

Table 1 shows the performance of the proposed model evaluated with different test images. The metrics used to estimate the performance of the proposed model are precision, recall, accuracy, and F1 score. The following are the equations for calculating the performance metrics.

(5) $$\begin{array}{c} \text{accuracy}=\frac{TN+TP}{Total}\times 100,\\ \text{precision}=\frac{TP}{TP+FP},\\ \text{recall}\left(\text{or}\right)\text{sensitivity}=\frac{TP}{TP+FN},\\ F1\text{ score}=\frac{2*\text{precision}*\text{recall}}{\text{precision}+\text{recall}}. \end{array}$$

**Figure 14:**
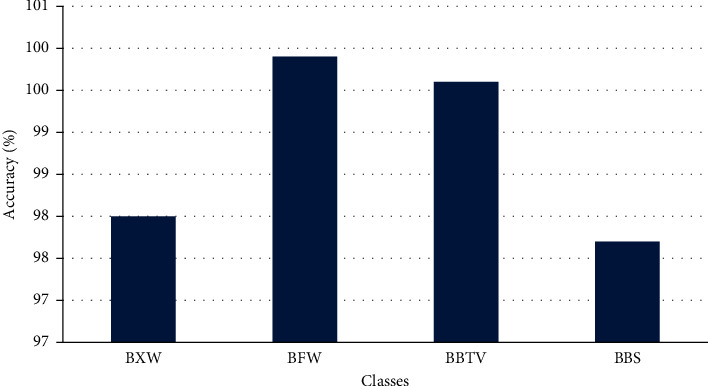
Accuracy of the classification.

**Figure 15:**
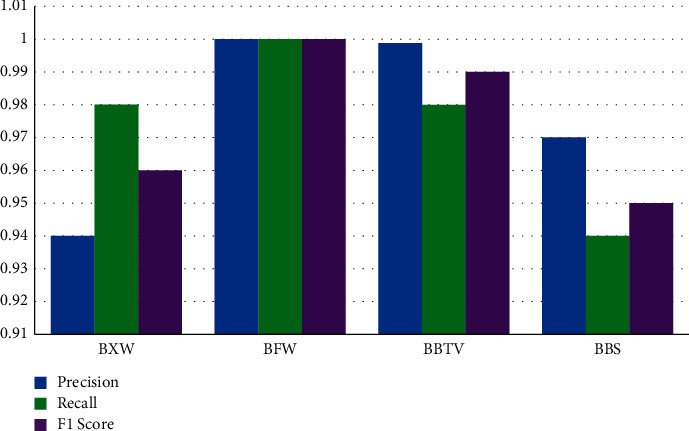
Performance evaluation.

Accuracy tells the overall correct prediction, the recall is the measure of positives that are misclassified as negative, precision is the measure of negatives that are misclassified as positives, F1 sore is the harmonic mean of precision and recall.  Figure 14 shows the accuracy levels of different classes predicted by the model, and Figure 15 shows the performance of other metrics.

**Figure 16:**
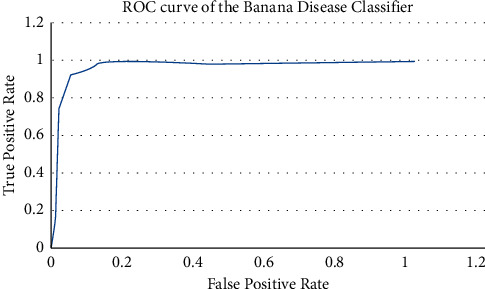
Receiver operating characteristic (ROC) curves for the proposed method.

**Figure 17:**
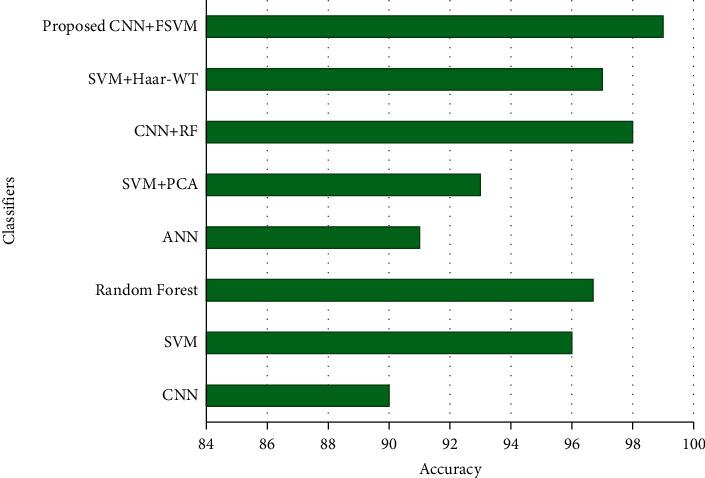
Comparison of various classifiers with the proposed method.

Figure 16 shows the ROC curves plotted between the true-positive rate and the false-positive rate for the proposed method, and Figure 17 shows the comparison of the proposed model with other classifiers based on its accuracy. This comparison was made based on the literature survey comparing various classifiers such as CNN, SVM, random forest, SVM + PCA, ANN, CNN + RF, and SVM + Haar-WT. Comparison results show that CNN + fusion SVM-based classifier accelerates performance for classifying multiple classes.

## 5. Conclusion and Future Scope

Agriculture is very significant in producing food for humanity and plays an important role in the growth of the country's economy. So the proper management of the agricultural products is very important. Banana is a very important and vital crop produced all over the world, and it is necessary to prevent bananas from getting infected due to harmful diseases such as banana Xanthomonas wilt (BXW), banana *Fusarium* wilt (BFW), banana bunchy top virus (BBTV) and banana black Sigatoka (BBS). In this research, an integrated deep learning-based solution has been proposed for detecting and classifying the banana disease by investigating not only the banana leaf but also with other parts of the plant with the help of CNN and an FSVM, which is a combination of binary and multiclass SVM. The proposed method shows an accelerating performance of an overall accuracy of 99%, with an excellent precision-recall and F1-score, which shows that the proposed method is best suitable for detecting the disease in the banana tree. The list of abbreviations is listed in Table 2.

The future work is to develop a system and implement the proposed system in such a way that the disease can be detected in banana cultivation in the agricultural farm with the aerial view drone camera instead of investigating an individual plant. So that the intensity of the disease spreading throughout the field can be easily estimated, which also helps in identifying the overall yield and loss in case of any disease infection, also furthermore a user-friendly mobile application will be created for the farmers to instantly identify the disease and react accordingly. [17–25].

## Figures and Tables

**Algorithm 1:**
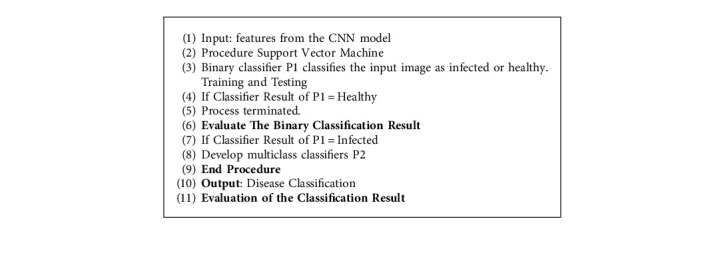
Proposed fusion SVM classifier.

**Table 1:** Performance evaluation of the proposed classification.

Class	F1 score	Accuracy (%)	Recall	Precision
BXW	0.96	98	0.98	0.94
BFW	1	99.90	1	1
BBTV	0.99	99.60	0.98	1
BBS	0.95	97.70	0.94	0.97

**Table 2:** List of abbreviations.

Abbreviation	Acronyms
CNN	Convolution neural network
FSVM	Fusion support vector machine
BXW	Banana Xanthomonas wilt
BFW	Banana fusarium wilt
BBTV	Banana bunchy top virus
BBS	Banana black Sigatoka
RGB	Red green blue
HSI	Hue, saturation, and intensity
PCA	Principle component analysis
HOT	Histogram of template
R–CNN	Region-based convolution neural network
ANN	Artificial neural network
LBPH	Local binary pattern histogram
Haar-WT	Haar-wavelet transform
AUC	Area under curve
KNN	K-nearest neighbors
VGA	Video graphics array
DSLR	Digital single-lens reflex
ReLU	Rectified linear unit
AMD	Advanced microdevices
GPU	Graphics processing unit
ET	Elapsed time
TP	True positive
TN	True negative
FP	False positive
FN	False negative
ROC	Receiver operating characteristic
RF	Random forest

## Data Availability

Data will be made available upon request
